# Automatic assessment of the cardiomyocyte development stages from confocal microscopy images using deep convolutional networks

**DOI:** 10.1371/journal.pone.0216720

**Published:** 2019-05-30

**Authors:** Pavel Škrabánek, Alexandra Zahradníková

**Affiliations:** 1 Institute of Automation and Computer Science, Brno University of Technology, Brno, Czech Republic; 2 Institute of Molecular Physiology and Genetics, Centre of Biosciences SAS, Bratislava, Slovakia; 3 Department of Cellular Cardiology, Inst. of Experimental Endocrinology, Biomedical Research Center SAS, Bratislava, Slovakia; Pennsylvania State Hershey College of Medicine, UNITED STATES

## Abstract

Computer assisted image acquisition techniques, including confocal microscopy, require efficient tools for an automatic sorting of vast amount of generated image data. The complexity of the classification process, absence of adequate tools, and insufficient amount of reference data has made the automated processing of images challenging. Mastering of this issue would allow implementation of statistical analysis in research areas such as in research on formation of t-tubules in cardiac myocytes. We developed a system aimed at automatic assessment of cardiomyocyte development stages (SAACS). The system classifies confocal images of cardiomyocytes with fluorescent dye stained sarcolemma. We based SAACS on a densely connected convolutional network (DenseNet) topology. We created a set of labelled source images, proposed an appropriate data augmentation technique and designed a class probability graph. We showed that the DenseNet topology, in combination with the augmentation technique is suitable for the given task, and that high-resolution images are instrumental for image categorization. SAACS, in combination with the automatic high-throughput confocal imaging, will allow application of statistical analysis in the research of the tubular system development or remodelling and loss.

## Introduction

The cardiac muscle cells, cardiomyocytes, contract to propel blood flow [1]. In adult heart, ventricular myocytes contain a system of membraneous transversal tubules (t-tubules) continual with the sarcolemmal membrane [2]. T-tubules form during postnatal growth and maturation of cardiac myocytes and are prone to remodelling under physiological or pathological cardiac hypertrophy [3]. As the integrity of the tubular system is essential for the correct function of adult cardiac myocytes, understanding of their formation, loss and remodelling, and factors influencing these processes is paramount for both the study of excitation-contraction coupling, as well as for the development of mature human induced pluripotent stem cell derived cardiomyocytes with correct phenotype [4].

Several developmental stages of t-tubule formation were identified in confocal microscopy images of fluorescently labelled sarcolemma of cardiomyocytes from growing rat hearts; however, high variability among myocytes of the same heart was observed and therefore the individual stages could not be simply assigned to specific period of heart development [5]. An alternative to quantification of t-tubules in single myocytes could be application of statistical methods on a large population of cardiomyocytes. This statistical evaluation would allow assessment of the t-tubule development, loss, and remodelling in different age groups or under different conditions. Current microscopy technologies, including confocal microscopy, enable automatic collection of large amounts of images; thus, allowing implementation of the statistical evaluation of t-tubule formation.

Different sampling modes are used in cardiomyocyte research while taking microscopy images. In one, the expert identifies and manually localizes individual myocytes in a field of view. The marked areas are then scanned [6]. Alternatively, signals are recorded at low spatial resolution and measured from the whole field of view. All obtained images may be later manually processed by the expert [7]. Both alternatives are suitable when a small number of target objects should be captured, e.g. when recording functional data from live cells, where several measurements are made from the same cell or culture plate well. Statistical analysis of morphological features, on the other hand, requires a high number of images of various individually recorded cells. These images can be easily obtained from the whole culture well or microscope slide of fixed cells using mosaic scanning functionality of modern confocal microscopes. Unfortunately, only a part of such created dataset contains high quality images of whole and healthy myocytes. The rest of the dataset consists of empty images, images of dead cardiomyocytes, out-of-focus images and images of cell fragments. Thus, before the data can be statistically analysed, the proper images in the datasets must be identified and sorted.

So far, when attempting to quantify the t-tubule complexity in cardiac myocytes, expert needs to identify healthy myocytes in the sample and record high quality images, which are then quantified using 2D spatial Fourier transform [8–11] or stereological analysis [5]. To statistically analyse the whole population, the expert would need to assign the t-tubule development stage to each observed image of a cardiomyocyte according to a complex set of object features and classification rules. Such an approach is impractical and prone to subjective errors. Considering the amount of data required for the statistical analysis, automatic classification of the images would be more appropriate. As the cardiomyocytes are objects of high complexity and show substantial morphological variability, the automatic classification is hindered by lack of implemented methods.

The automatic classification of images is a typical computer vision task known as generic object categorization [12]. The state-of-the-art image categorization systems rely on deep convolutional networks (deep ConvNets) [13]. Deep ConvNets naturally integrate feature extraction and classification into one compact unit. Before their utilization, they must be trained and evaluated on sets of labelled samples. Key factors influencing performance of a trained deep ConvNet-based image categorization system are the quality of the used training set and a learning capacity of the network.

The quality of a training set is mainly influenced by selection of training samples, their correct categorization, and the total number of samples of each category in the training set [14]. A class balance issue must be also considered whenever class-sensitive learning method is used [15]. In microscopy image analysis, a limited amount of source data is usually available [16–18] which is also the case of confocal microscopy images of the cardiomyocytes. The lack of data limits sample selection and does not allow creation of a sufficiently large and representative training set. To overcome these concerns, data augmentation techniques, such as image translations, horizontal reflections [17], or rotations [16, 18] are typically applied on inadequate sets. The representativeness of the sets can be further improved by using images independently categorized by several experts.

The capacity of a deep ConvNet is predetermined by its topology. A typical topology of the early deep ConvNets is as follows. The first levels of a network consist of convolutional layers, typically complemented with rectified linear unit activation functions, and of several pooling layers [13]. Subsequent levels usually contain fully connected layers including a dropout regularization technique [19–21]. The network is typically closed by a classifier employing a softmax function. The layers in the network are arranged in a feed-forward manner and the capacity of the network can be increased by increasing the number of layers (a depth of the network) [13]. Such a topology, however, does not allow construction of very deep networks due to a vanishing gradient problem [22]. Thus, deep ConvNets based on this topology are appropriate for problems with a relatively simple discriminability of target objects, e.g. for human epithelial-2 cell image classification [16], for classification of glioblastoma multiform and low-grade glioma [17], or for classification of red blood cells in sickle cell anemia [23]. This topology is, however, inappropriate for the classification of cardiomyocyte images due the complexity of the cardiomyocytes.

Modern, more complex deep ConvNet topologies overcome the vanishing gradient problem to a large extent; hence, they allow construction of very deep networks with great learning capacities. The new topologies control the capacity by varying width or depth of networks [21]. Enlarging a deep ConvNet capacity through increasing its width is used e.g. in GoogLeNet [24, 25], where several sub-networks are connected in parallel at various levels of the network. The current trends converge towards increased number of layers (the network depth), while retaining the data processing linearity. Topologies, such as Highway Networks [26], Residual Networks [27–29], Deep Pyramidal Residual Networks [30], Densely Connected Convolutional Networks (DenseNets) [31] and Cross-Layer Neurons Networks [32], fall into this category. All these topologies show very good classification performance even on datasets with high intraclass variability.

Given the progress in computer vision in last years, we decided to develop a system for automatic assessment of cardiomyocyte development stages (SAACS) from confocal images. SAACS is aimed to identify and classify whole and healthy myocytes in a set of images obtained while scanning the whole culture well or microscope slide of fixed cells, where the images are classified according to the developmental stages of individual cardiomyocytes. Considering the complexity of the cardiomyocytes and the limited number of images available for the forming of the training set, we based SAACS on the DenseNet topology. As the other modern deep ConvNet topologies, DenseNet topology alleviates the vanishing-gradient problem and it allows creation of networks with high learning capacity. Furthermore, according to [31], networks of DenseNet topology are proved to be robust against overfitting on tasks with small training sets. For a further improvement of the training process, we designed an efficient data augmentation technique that improved classification performance of SAACS. We show that SAACS trained on the augmented dataset, consisting of fifteen source images of each class only, was able to identify the whole and healthy myocytes, and differentiate among five cardiomyocyte development stages. In addition, we analysed the influence of image resolution on SAACS performance and implemented a class probability graph to simplify the expert evaluation of SAACS performance.

## Materials and methods

### Isolated cardiomyocytes

#### Ethical statement

Animals were housed and treated according to the European directive for the protection of animals used for scientific purposes (2010/63/EU) and with the Laboratory Animals Act No. 377/2012 and the Decree 436/2012 Z.z. SR. All procedures were approved by the State Veterinary and Food Administration of the Slovak Republic (3514/14-221) and by the Ethical committee of the Institute of Molecular Physiology and Genetics, Slovak Academy of Sciences. Adult males and pregnant females were from Dobra Voda, Slovakia.

#### Confocal microscopy

Sarcolemma of isolated cardiomyocytes was stained using lipophilic membrane probes (di-8-ANEPPS or FM4-64, Molecular probes, Oregon, US). Images were obtained by the Leica TCS SP2 AOBS confocal microscope (Leica Microsystems, Germany) equipped with HCX PL APO CS 63x/1.2 NA water immersion objective. Fluorophores were excited by 488 nm and combined 496 and 514 nm laser light. Fluorescence emission was collected from 520 to 800 or 620 nm to 760 nm windows for di-8-ANEPPS or FM 4-64, respectively. Images were recorded in x-y mode with 58 nm to 116 nm per pixel (px) with 4× frame averaging to increase the signal-to-noise ratio, with the confocal aperture set to 1 Airy unit.

### Training and evaluation sets

#### Classification of object images

The expert identified the live and healthy cardiomyocytes in images and assigned them into 5 development stages. The least developed cardiomyocytes were considered to be at stage 1 while the most developed ones were assigned stage 5 (Table 1). We used the stages to define classes of object images where the stages 1 to 5 correspond to classes 1 to 5, respectively. The rest of images in the collection (low quality images or images which do not contain whole and healthy myocytes) belong to the class 0.

**Table 1:** Characterization of cardiomyocyte development stages. Experts assess cardiomyocyte development stages according to patterns of longitudinal and transversal tubules, considering their quantity and character, and according to the complexity of the tubular system.

stage	longitudinal tubules		transversal tubules		system complexity
	quantity	character	quantity	character	
1	none	-	none	-	no
2	low	long	low	short	no—very low
3	high	long	medium	medium	low—medium
4	medium	medium	high	long	high
5	low	short	high	long	high

**Fig 1:**
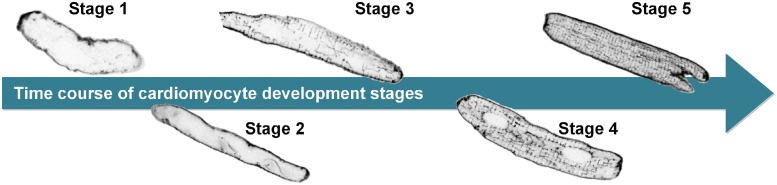
Development stages of cardiomyocytes. The images of cardiomyocytes are ordered from left to right according to their developmental stages, from the least to the most developed (stage 1 to stage 5).

Cardiomyocytes with no membrane invaginations were categorized as stage 1. Stage 2 was characterized by presence of short (~2 μm), regularly spaced, perpendicular invaginations of the membrane and/or individual long (>10 μm) tubules. In stage 3, a web of longer tubules (>10 μm), both transverse and longitudinal, was present. A complex system of transversal tubules with manifest presence of longer (>2 μm) longitudinal tubules and frequent areas devoid of tubules was classified as stage 4. Stage 5, present in adult cardiomyocytes, was distinguished by a complex system of transversal tubules, with sparse longitudinal structures spanning usually not more than 1 to 2 sarcomeres (~2 μm to ~4 μm) filling the whole area of the cell image except nuclei. Examples of development stage categories are shown in Fig 1.

#### Source images

**Fig 2:**
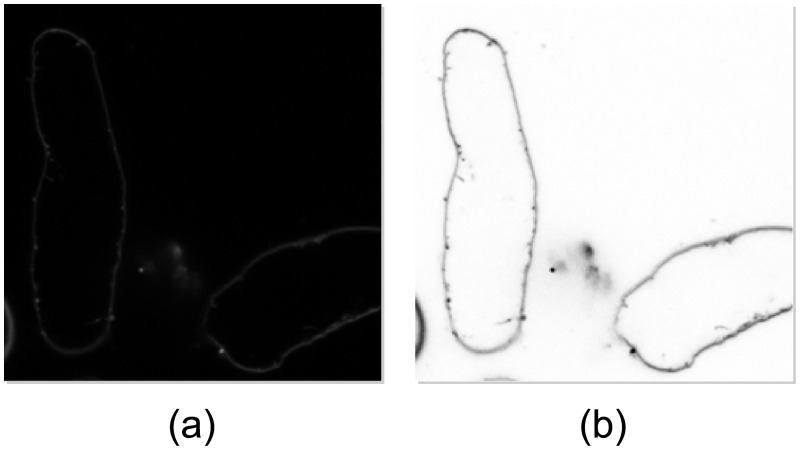
Example of a source image. The source images are monochromatic images of spatial resolution 1024 × 1024 px obtained using the confocal microscopy. Objects in source images (a) are poorly visible. Within this article, we use images enhanced using inversion and gamma correction (b).

Confocal microscopy produces high-resolution monochromatic images of optional dimensions. In our case, images 1024 × 1024 px were considered and each image contained not more than one target object (cardiomyocyte). An example of original source image and of its enhancement for better representation in the article are shown in Fig 2. As the images were recorded in the traditional way (manually by an expert), a very limited number of appropriate images was available to form a training and an evaluation set. Our dataset consisted of the images analysed in [5], to which we added 12 images of class 5, and all class 0 images. In total, 18 images of class 1, 23 images of class 2, 22 images of class 3, 22 images of class 4, and 26 images of class 5 were available. Compared to classes 1 to 5, the number of available class 0 images was not limiting. Our collection of source images was imbalanced, which might compromise training of deep ConvNets [33]. To deal with the imbalanced data issue, a random under-sampling method [34] was used to form a class-balanced set of source images. To create the set, we randomly selected 15 images of each class from the original image collection. The class-balanced set was used as the base for creating the training set. The remaining (unselected) images were used as source images for the evaluation set. In addition, 15 and 9 source images of class 0 were allocated to the training and the evaluation set, respectively. The data are available at [35].

#### Data augmentation

The small number of source images did not allow efficient training (90 images) or credible evaluation (46 images) of any deep ConvNet [13]. To overcome this issue, data augmentation techniques were used. To prevent alteration of t-tubule transversal pattern, which we consider important for correct classification, non-destructive augmentation techniques were chosen. Specifically, the source images were horizontally flipped, and both the flipped and the original images were rotated by an angle *φ* ∈ {0, Δ*φ*, 2Δ*φ*, …, 2*π*}, where $\Delta \varphi =\frac{2\pi }{144}$ (first method).

**Fig 3:**
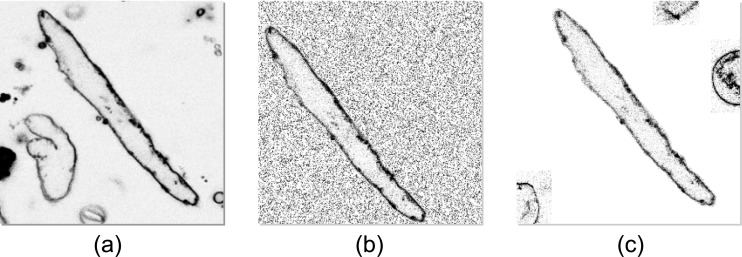
Augmentation of source images by manually modified images. A cut-out of a cardiomyocyte was created from the original source image (a). Placing the cardiomyocyte on a noisy and neutral background, two manually modified source images (b) and (c) were created, respectively. The second manually modified image (c) was enhanced by cell fragments and out-of-focus cardiomyocytes. Note that the displayed images were inverted and enhanced using gamma correction for better representation in the article.

Additionally, to enhance the recognition of the objects of interest, we have developed a second augmentation technique, which applies the first method on an extended set of source images. The extended set consisted of the original source images and manually modified images. The modified images were based on cut-outs of target objects (cardiomyocytes). From each original source image (Fig 3(a)), the target object was extracted; and from each extraction, two new source images were created. In both images, the target object was placed on a neutral or noisy background, at a random position within the image. While the first new image consisted only of the target object and the background (Fig 3(b)), the second one (Fig 3(c)) was enhanced with non-target objects that naturally occur in the confocal images. The non-target objects were placed randomly in the image, but they did not overlap with the target object. In this way, the source images of classes 1 to 5 were extended. Class 0 images were extended by 30 new unique source images. Thus, the set augmented using this technique was three times larger than the set produced by the first data augmentation technique.

#### Set up of training and evaluation sets

We hypothesized that due to the small size (~0.1 μm to ~0.3 μm in diameter) and dense spacing (~1.8 μm to ~2.0 μm) of t-tubules, high-resolution images are key for the efficient assessment of the cardiomyocyte development stages. The available source images were of dimensions 1024 × 1024 px, with resolution 58 nm to 116 nm per px. To assess the importance of image resolution on SAACS classification performance, we created low-resolution sets of source images. The original source images were resized to 512 × 512 px using a resize method with bicubic resampling filter in python imaging library 5.0.0, resulting in resolution of 116 nm to 232 nm per px.

We applied both augmentation techniques on the resized (512 × 512 px) and the original (1024 × 1024 px) source images. Using the first augmentation technique (A), we created training sets T-512-A and T-1024-A, and evaluation sets E-512-A and E-1024-A. Using the second augmentation technique (B), we formed training sets T-512-B and T-1024-B. The training sets T-512-A and T-1024-A consisted of 25 920 labelled object images, and the evaluation sets E-512-A and E-1024-A consisted of 13 248 labelled object images. The training sets T-512-B and T-1024-B consisted of 77 760 labelled object images.

### SAACS

#### DenseNets

DenseNets are deep ConvNets with an advanced topology. In addition to commonly used convolutional, pooling and fully connected layers [13], two composite building elements, *dense blocks* (DBs) and *transition layers* (TLs), are used to create a DenseNet [31].

#### Dense block

**Fig 4:**
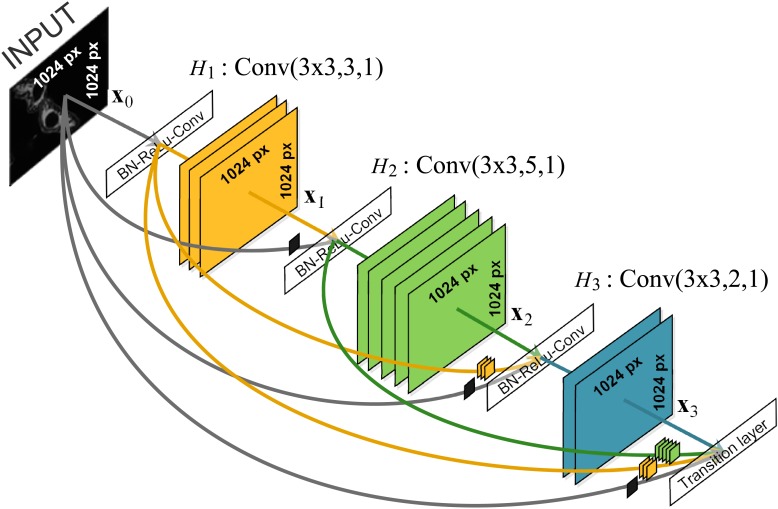
Layout of a general dense block. A dense block consists of *d* layers where the layers are connected using concatenation and transformed using non-linear transformations *H*(⋅). Typically, a transition layer follows a dense block. The figure shows a hypothetical dense block of three layers (*d* = 3) which is placed at the input of a network. Non-linear transformations *H*(⋅) within this block are composite functions which consist of the batch normalization (BN), the rectified linear unit (ReLU) and the convolution (Conv), respectively. At the first, second and third layer, three, five and two convolutional filters (*f*_1_ = 3, *f*_2_ = 5, *f*_3_ = 2) of height and width 3 × 3 px (*h* = *w* = 3) convolve with stride one (s = 1). The input data of the network **x**_0_ is a confocal microscopy image of resolution 1024 × 1024 px. At the first, second and third layer of the dense block, feature maps **x**_1_, **x**_2_ and **x**_3_ of depth three, five and two originate, respectively. Spatial resolutions of the feature maps are 1024 × 1024 px. Input of each layer is composed of feature maps that arose on the preceding layers of the dense block, and of the input of the block (the confocal microscopy image **x**_0_ in this case). The block input data and the feature maps are catenated at the input of each layer.

A DB consists of several layers connected in a dense pattern where each layer takes all preceding feature-maps as its input (Fig 4). The layers are connected using concatenation and transformed using a non-linear transformation. Let us consider a *n*-th DB that is built in a DenseNet of *L* layers. A non-linear transformation performed within this DB, placed at the *ℓ*-th level within the network, produces feature maps **x**_*ℓ*_ that are given as

$$\begin{array}{c} x_{\ell }=H_{\ell }\left(\left[x_{i_{n}},x_{i_{n}+1},\ldots ,x_{\ell -1}\right]\right), \end{array}$$ (1)

where *H*_*ℓ*_(⋅) is the *ℓ*-th non-linear transformation, $\left[x_{i_{n}},x_{i_{n}+1},\ldots ,x_{\ell -1}\right]$ refers to the concatenation of the feature maps **x** produced in layers *i*_*n*_, …, (*ℓ* − 1). Note that $x_{i_{n}}$ are feature maps fed into the input of the *n*-th DB. The input of the DB is placed at the *i*_*n*_-th level of the network, *i*_*n*_ < *ℓ* ≤ *o*_*n*_, and *o*_*n*_ is a level at which the output of the *n*-th DB is placed.

The *ℓ*-th non-linear transformation *H*_*ℓ*_(⋅) is a composite function of several consecutive operations. We used a basic and a bottleneck version of the composite function [31]. The basic version consists of three operations: batch normalization (BN) [36], followed by a rectified linear unit (ReLU), followed by a convolution (Conv) [13]. Using a short notation, this version of the function can be written as BN-ReLU-Conv(*h* × *w*, *f*, *s*), where *s* is stride of convolutional filters, *f* is number of the filters, and *h* and *w* are their height and width, respectively. The bottleneck version of the composite function is defined as BN-ReLU-Conv(1 × 1, 4*f*, 1)-BN-ReLU-Conv(*h* × *w*, *f*, *s*). If necessary, convolutions were zero-padded to keep the feature-map size fixed.

Each DB consists of *d* layers either with the basic or with the bottleneck version of the composite function. For the *n*-th DB, it holds that *d*_*n*_ = *o*_*n*_ − *i*_*n*_. For both versions of the composite function, the parameters *h*, *w*, *s*, *f* are identical for all layers within a DB. DBs with basic and bottleneck version of the function will be denoted as DBa(*h* × *w*, *s*, *f*, *d*) and DBb(*h* × *w*, *s*, *f*, *d*), respectively.

#### Transition layer

**Fig 5:**
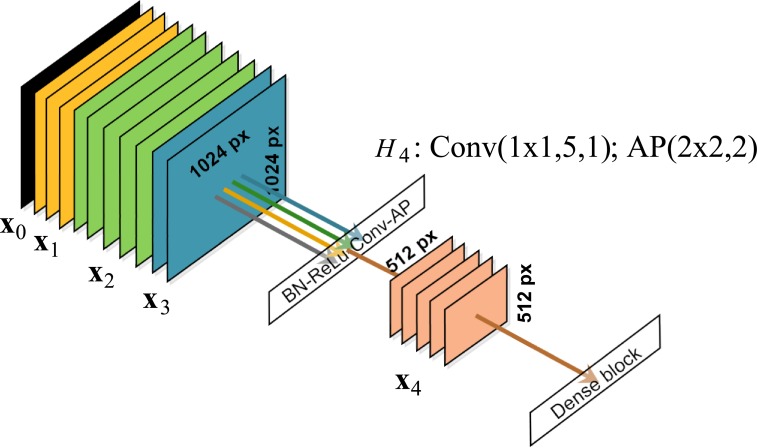
Layout of a transition layer. Primary, transition layers reduce spatial resolution of the feature maps **x** to reduce the number of parameters of the network. They are placed behind dense blocks. In this case, the transition layer is placed behind the dense block from Fig 4. The input of the network **x**_0_ and the feature maps **x**_1_, **x**_2_, **x**_3_ are catenated and processed by the non-linear transformations *H*_4_(⋅). The transformation is a composite function which consists of the batch normalization (BN), the rectified linear unit (ReLU), the convolution (Conv) and the average pooling (AP), respectively. Five convolution filters (*f* = 5) of height and width 1 × 1 px (*h* = *w* = 1) with stride one (s = 1) are used. The number of the convolution filters *f* controls the number of feature maps produced by the transition layer, i.e. depth of the feature maps **x**_4_ is five in this case. Pools 2 × 2 px and stride 2 are used in AP. Thus, the original data of spatial resolution 1024 × 1024 px are reduced to 512 × 512 px.

Let us consider a TL connected at the output of the *n*-th DB (i.e. the layer is placed at the (*o*_*n*_ + 1)-th level of the network). Feature maps produced by this layer are given as

$$\begin{array}{c} x_{o_{n}+1}=H_{o_{n}+1}\left(\left[x_{i_{n}},x_{i_{n}+1},\ldots ,x_{o_{n}}\right]\right), \end{array}$$ (2)

where $\left[x_{i_{n}},x_{i_{n}+1},\ldots ,x_{o_{n}}\right]$ denotes the concatenation of all feature maps that appear in the *n*-th DB. The non-linear transformation $H_{o_{n}+1}$ is also a composite function. The function was defined as BN-ReLU-Conv(1 × 1, *f*, 1)-AP(2 × 2, 2), where AP(2 × 2, 2) denotes an average pooling with pools 2 × 2 and stride 2 [31]. The function of transition layers is demonstrated in Fig 5.

The 1 × 1 convolution incorporated in the TL allows improvement of network compactness. The number of convolutional filters *f* controls the number of feature maps produced by the TL. Considering that the *n*-th DB produces *m*_*n*_ feature maps, the number of feature maps produced by the (*o*_*n*_ + 1)-th TL is given as $f_{o_{n}}{}_{+1}=\left\lfloor \theta m_{n}\right\rfloor$, where *θ* is a compression factor, and $\theta \in [m_{n}^{-1},1]$.

#### SAACS structure

SAACS is a DenseNet that performs feature extraction and classification on size-normalized object images. We designed and implemented two variants of SAACS (i.e. two DenseNets). One variant (DenseNet-512) was aimed at object images of dimensions 512 × 512 px and the second one (DenseNet-1024) at object images of dimensions 1024 × 1024 px. As the object images are monochromatic, numbers of input channels of the networks (depths of the network inputs) are one.

Both the DenseNet-512 (Table 2) and the DenseNet-1024 (Table 3) are opened by one DBa followed by a max pooling layer (MPL). The DBa consists of one layer (*d* = 1) with 2*k* convolutional filters (*f* = 2*k*) with kernels of size 7 × 7 px (*h* = *w* = 7), stride by 2 px (*s* = 2). The variable *k* is a hyperparameter determining the number of filters *f* in all DBs within the network. We used *k* = 20 and *k* = 15 for DenseNet-512 and DenseNet-1024, respectively. At the MPL, 3 × 3 px (*h* = *w* = 3) pools stride by 2 (*s* = 2) were used.

**Table 2:** Structure of the DenseNet-512 classifying 512 × 512 px confocal microscopy images of cardiomyocytes into development stages.

	DBa	MPL	DBb	TL	DBb	TL	DBb	TL	DBb	TL	DBb	GAP	C
*h*	7	3	3	-	3	-	3	-	3	-	3	7	-
*w*	7	3	3	-	3	-	3	-	3	-	3	7	-
*s*	2	2	1	-	1	-	1	-	1	-	1	7	-
*f*	2*k*	-	*k*	-	*k*	-	*k*	-	*k*	-	*k*	-	-
*d*	1	-	6	-	9	-	12	-	15	-	18	-	-

In the first row, the used building components are listed with respect to their placement in the network (the first block is the leftmost one); where DBa and DBb are the basic and the bottleneck versions of the dense blocks; MPL is the max pooling layer; TL is the transition layer, GAP denotes the global average pooling, and C is used for a classifier that consists of one fully connected layer followed by the softmax function. The parameters *h* and *w* are the height and weight of the filter kernel or of the pool; *s* is stride of the kernel of the pool; *f* is the number of filters at one convolution in the dense block; and *d* is the number of layers in the dense block.

**Table 3:** Structure of the DenseNet-1024 classifying 1024 × 1024 px confocal microscopy images of cardiomyocytes into development stages.

	DBa	MPL	DBb	TL	DBb	TL	DBb	TL	DBb	TL	DBb	TL	DBb	GAP	C
*h*	7	3	3	-	3	-	3	-	3	-	3	-	3	7	-
*w*	7	3	3	-	3	-	3	-	3	-	3	-	3	7	-
*s*	2	2	1	-	1	-	1	-	1	-	1	-	1	7	-
*f*	2*k*	-	*k*	-	*k*	-	*k*	-	*k*	-	*k*	-	*k*	-	-
*d*	1	-	6	-	8	-	8	-	10	-	15	-	15	-	-

The meaning of the symbols and variables is explained in Table 2.

The inner parts of the networks consist of several DBbs where each DBb is followed by one TL. In DenseNet-512, 4 DBbs are used. They have 6, 9, 12 and 15 layers, respectively. In DenseNet-1024, 5 DBbs are incorporated. They have 6, 8, 8, 10, and 15 layers, respectively. Both the DenseNet-512 and the DenseNet-1024 are closed by one additional DBb, followed by a global average pooling (GAP) and a classifier. The classifier consists of one fully connected layer of six neurons followed by the softmax function. The number of layers within the closing DBb is 18 and 15 for the DenseNet-512 and the DenseNet-1024, respectively. In both variants of DenseNets, kernels of size 3 × 3 px stride by 1 px are used in all DBbs, and *f* = *k* for all of them. The compression factor *θ* = 0.5 is used for both networks. Structures of both networks and their parameters were determined based on a pilot study.

### SAACS evaluation

For the SAACS performance evaluation, we used an average per-class accuracy ($\bar{\text{acc}}$), a macro-averaging precision (pr_*M*_), and a macro-averaging recall (re_*M*_) [37]. These standard measures are sufficiently informative if the evaluation is performed on a set with non-overlapping classes.

As the growth of cardiomyocytes and t-tubules is a continuous process, an individual myocyte might possess a combination of features belonging to different neighbouring classes (i.e. the classes may overlap). It means that both the expert assessments and the SAACS classifications may vary, which negatively influences the information value of the performance measures. Thus, we designed a class probability graph for a more precise evaluation of the SAACS performance.

SAACS classifies object images presented on its input. For an object image, the softmax function produces class label probability scores. The class probability graph displays scores of each sample (object image) in an evaluation set as a bar of fixed height. Starting with class 0, the bar is coloured from the top down, respecting the order of the classes. We organized samples in the evaluation set with respect to source images, their rotation and flipping. The bars (samples) are divided into non-flipped and flipped columns.

**Fig 6:**
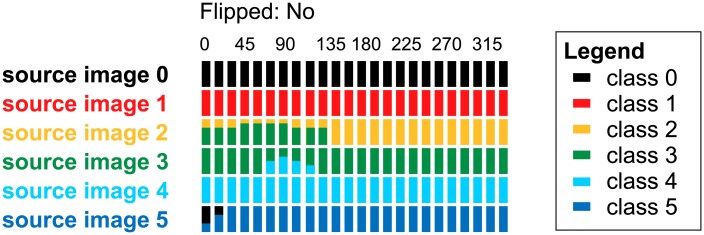
Class probability graph. The class probability graph displays label probability scores of 144 samples that are based on 6 source images. The samples were created by rotation of the source images about $\Delta \varphi =\frac{2\pi }{23}$. Rotations of the samples are shown at the first line (in degrees). Each class is associated with one colour. The class of the source image is highlighted using the appropriate colour. The assigned probability scores of the sample are displayed using the coloured bar.

Simply, Fig 6 depicts hypothetical evaluation results obtained on a set of 24 rotations (by $\Delta \varphi =\frac{2\pi }{23}$) of 6 source images, one of each class. The classes 0 to 5 are associated with black, red, yellow, green, light blue and dark blue colour, respectively. The source images are sorted in ascending order according to their class and displayed from top to bottom. The samples are sorted in ascending order according to their rotation angle *φ* (the angle in degrees is stated above the first row of bars).

In this example, all samples of the classes 0, 1 and 4 are correctly classified. The first 10 samples created from the source image 2 (class 2) were incorrectly classified as class 3. Similarly, the 7-th sample based on the source image 3 (class 3) was incorrectly classified as class 4. Such errors are questionable (classes 2 and 3, or classes 3 and 4 are neighbour classes), and should be reviewed by an expert. However, the incorrect assignment of the 1-st sample based on the source image 5 (class 5) is unjustifiable (classes 5 and 0 are not neighbours).

### SAACS implementation

The DenseNet-512 and the DenseNet-1024 were trained using an ADAM optimizer [38]. The learning rate *α* was set up at 5 ⋅ 10^−4^, and the exponential decay rate for the first and second moment estimates *β*_1_ and *β*_2_ were setup at 0.95 and 0.999, respectively. The optimizer and setting of the hyperparameters were determined based on a pilot study. A cross entropy function was minimized. The DenseNet-512 was trained on the sets T-512-A and T-512-B. The DenseNet-1024 was trained on the sets T-1024-A and T-1024-B. We used batches of size 9 and 3 samples while training the DenseNet-512 and the DenseNet-1024, respectively. Size of the batches was given by the capabilities of a used hardware. To ensure comparability of the results, the networks were trained on the same total number of samples (i.e. the same total number of iterations was carried out). Specifically, the networks were trained for 15 epochs on T-512-A and T-1024-A, and for 5 epochs on T-512-B and T-1024-B.

To keep comparability of the results, the DenseNet-512 trained on T-512-A or T-512-B was evaluated on the set E-512-A. Similarly, the DenseNet-1024 trained on T-1024-A or T-1024-B was evaluated on the set E-1024-A. Performance of the trained networks was assessed using $\bar{\text{acc}}$, pr_*M*_ and re_*M*_. Further, class probability graphs were used for expert assessment of SAACS performance.

We trained and evaluated the networks on a computer with 64 GB RAM, Ryzen 5 1600 CPU, and a graphic card nVidia GTX-1050 Ti with 4 GB GDDR5. The batches were formed from a shuffled queue of training samples. The training and evaluation of the networks was carried out in a python 3.6.4 environment using Tensorflow 1.5 [39]. Python implementations of the networks, of training and evaluation routines, likewise of a code which delineates class probability graphs are provided in a supplementary file S1 File.

## Results

The ability of SAACS to assess cardiomyocyte development stages from confocal microscopy images varied with respect to the composition of training sets and with respect to the resolution of object images (Table 4). The DenseNet-512 (variant of SAACS aimed at 512 × 512 px object images) trained on T-512-B (larger training set) showed better average per-class accuracy ($\bar{\text{acc}}=51.97\;\%$), macro-averaging precision (pr_*M*_ = 68.74%), and macro-averaging recall (re_*M*_ = 45.16%); compared to the DenseNet-512 trained on T-512-A ($\bar{\text{acc}}=42.72\;\%,{\text{pr}}_{M}=53.35\;\%,{\text{re}}_{M}=41.33\;\%$). The same trend could be observed in the results obtained by the DenseNet-1024 (SAACS processing object images of dimensions 1024 × 1024 px). We obtained $\bar{\text{acc}}=79.67\;\%,{\text{pr}}_{M}=72.08\;\%,{\text{re}}_{M}=71.29\;\%$ for the DenseNet-1024 trained on T-1024-B, and $\bar{\text{acc}}=64.98\;\%,{\text{pr}}_{M}=63.88\;\%,{\text{re}}_{M}=64.01\;\%$ for the network trained on T-1024-A. Average evaluation time was 0.46 and 0.54 seconds per image for the DenseNet-512 and DenseNet-1024, respectively.

**Table 4:** Evaluation results.

network training set	DenseNet-512		DenseNet-1024	
	T-512-A	T-512-B	T-1024-A	T-1024-B
$\bar{\text{acc}}$	42.72%	51.97%	64.98%	79.67%
pr_*M*_	53.35%	68.74%	63.88%	72.08%
re_*M*_	41.33%	45.16%	64.01%	71.29%

The DenseNet-512 and the DenseNet-1024 were trained on sets created using the first (T-512-A and T-1024-A) and the second data augmentation technique (T-512-B and T-1024-B). The trained networks were evaluated using average per-class accuracy ($\bar{\text{acc}}$), macro-averaging precision (pr_*M*_), and macro-averaging recall (re_*M*_). To provide comparable results, the same total number of iterations was carried out while training the networks.

**Fig 7:**
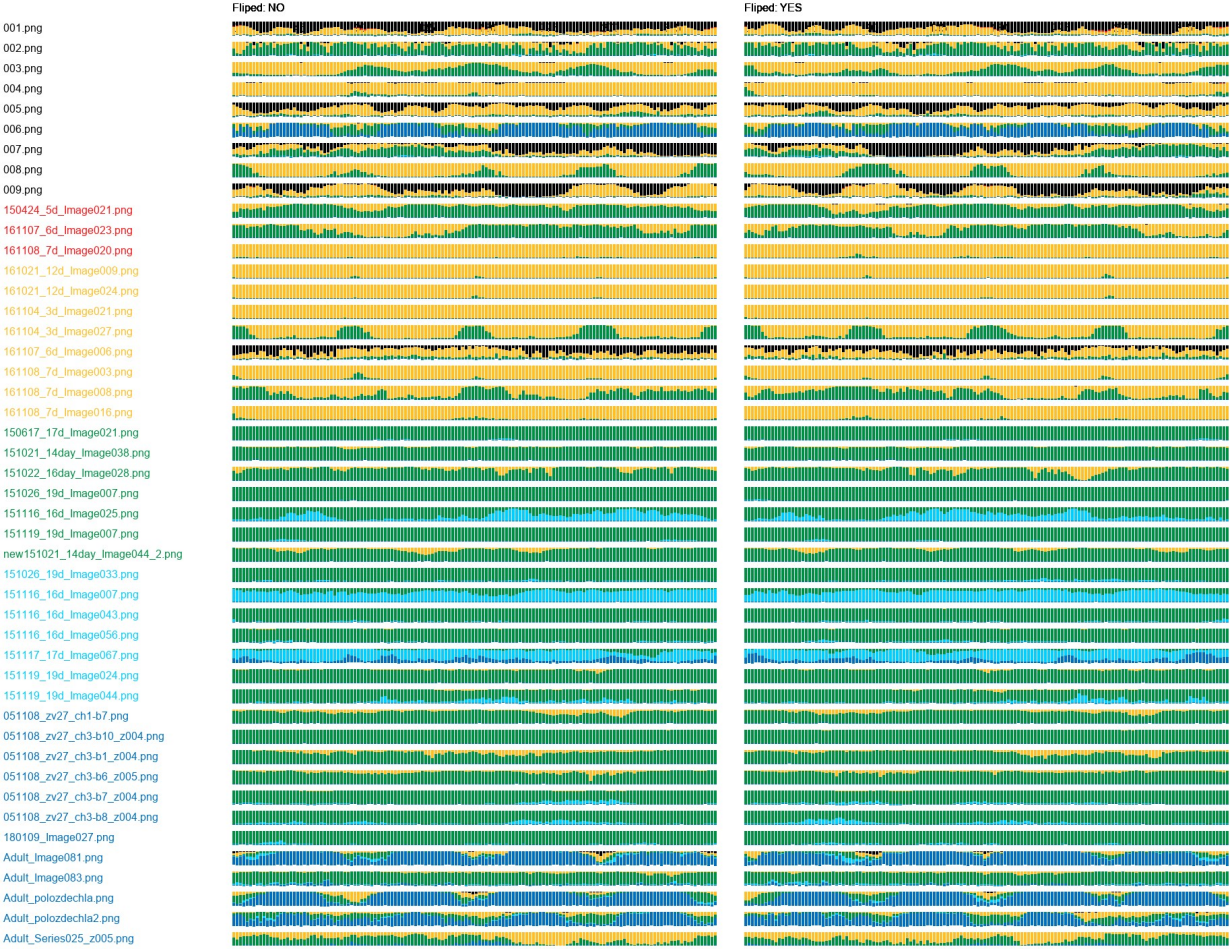
Class probability graph obtained for the DenseNet-512 trained on T-512-A. The trained network assigns most of the samples either to the class 2 or 3. Classes 0, 1 and 5 not recognized at all.

**Fig 8:**
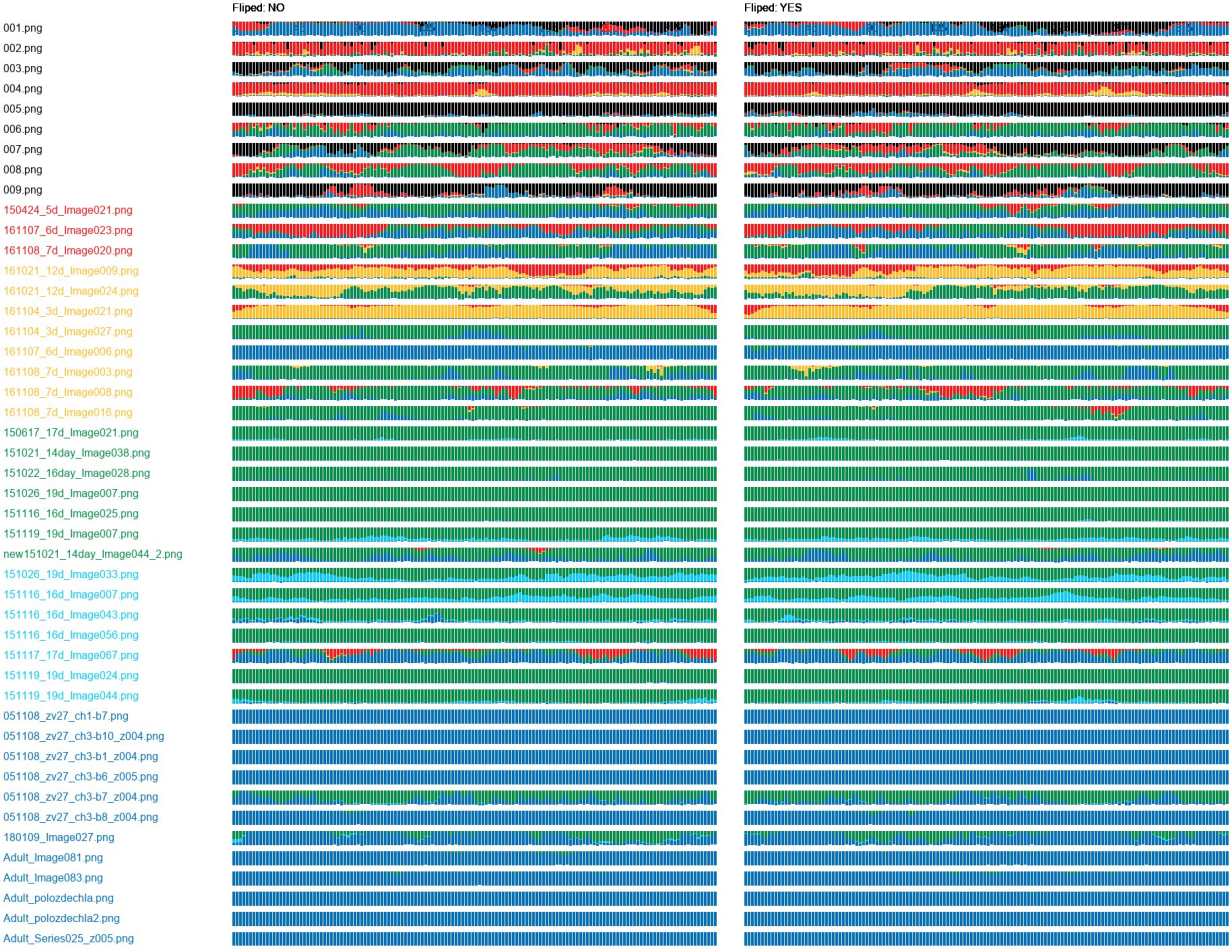
Class probability graph obtained for the DenseNet-512 trained on T-512-B. The trained network almost correctly recognizes the class 5 but same samples of other classes are also labelled as class 5. The network tends to label samples of other classes as the class 3; however, some samples of the classes 0 to 4 are correctly recognized.

**Fig 9:**
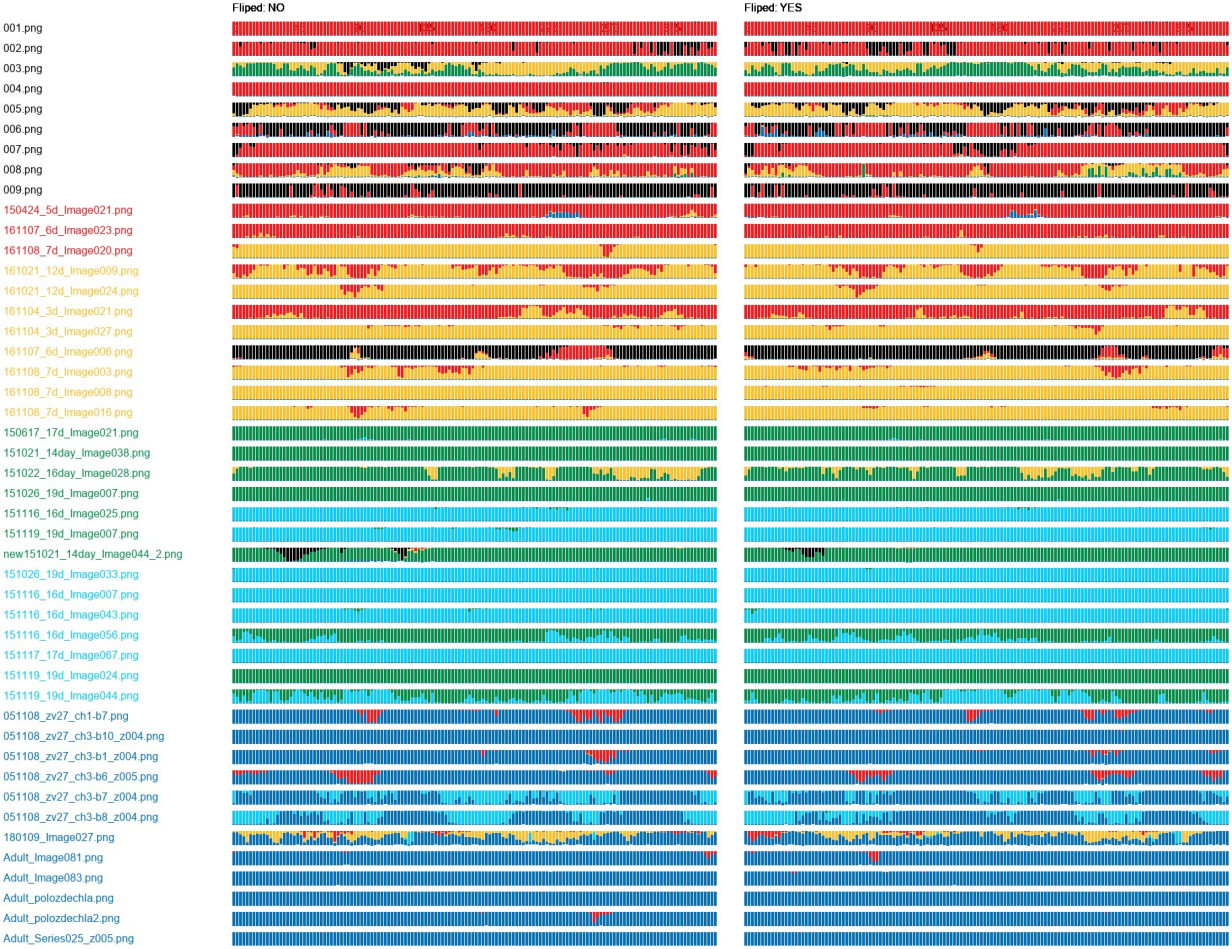
Class probability graph obtained for the DenseNet-1024 trained on T-1024-A. Except the class 0, the network correctly recognizes more than 50% of samples in each class; however, the 50% border line is exceeded only slightly for most of the classes. The only exception is the class 5 where almost all samples are correctly categorized.

**Fig 10:**
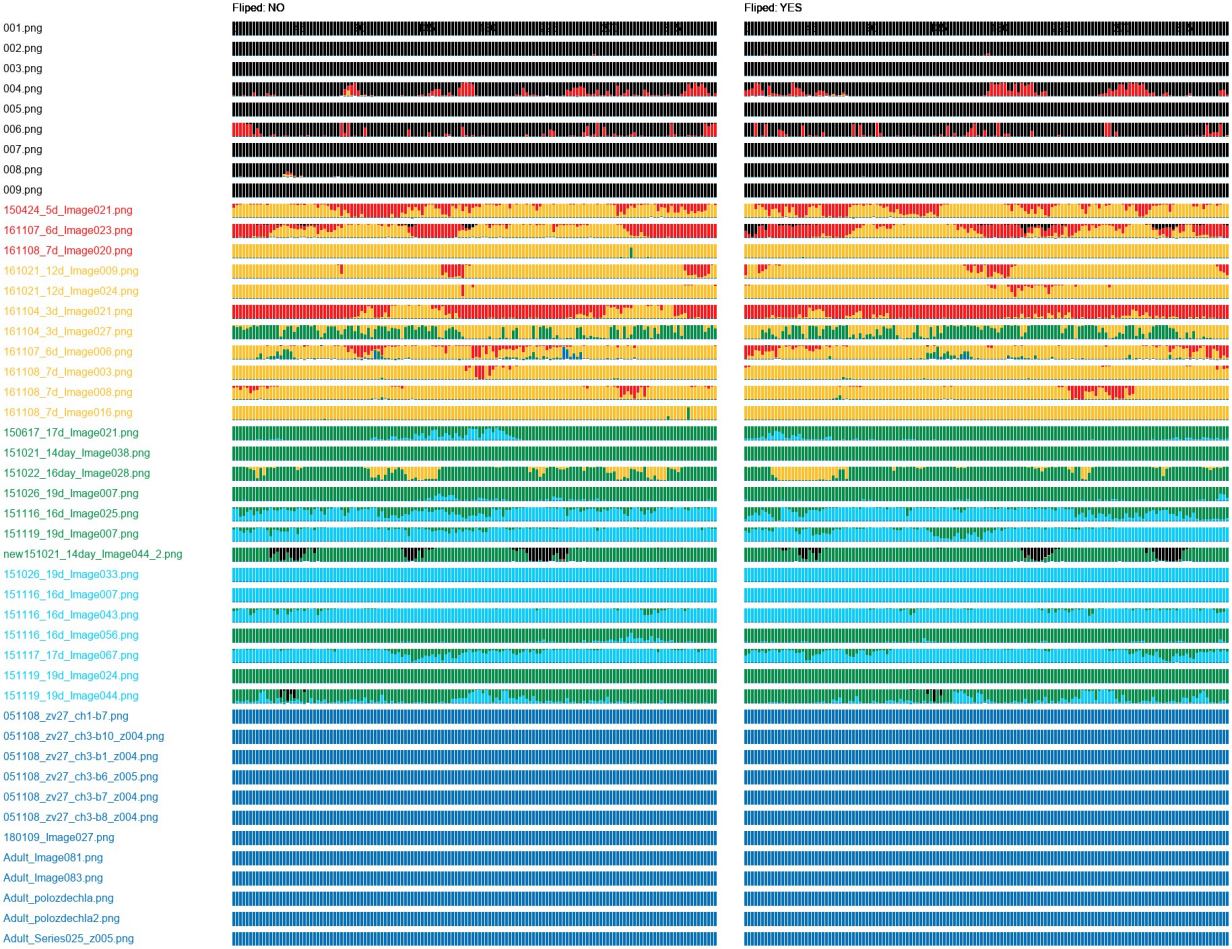
Class probability graph obtained for the DenseNet-1024 trained on T-1024-B. The trained network perfectly recognizes samples of the class 5. It does also very well on samples of the class 0. Some samples of other categories are incorrectly classifieds, but they are mostly assigned into neighbour classes.

For each combination of network and training set, we obtained one class probability graph. The graphs obtained for the DenseNet-512 (Figs 7 and 8) showed incapability of this network to recognize samples of the classes 0 and 1. The DenseNet-512 trained on T-512-A labelled most of the samples as class 2 or 3, even if they belonged to the class 5 (Fig 7). The same network trained on T-512-B tended to classify the samples as the classes 3 or 5. In this case, incorrectly classified samples were more often assigned to neighbour classes (Fig 8). Compared to the DenseNet-512, the DenseNet-1024 trained on T-1024-A was more accurate in classification of samples from classes 1 to 5 (Fig 9). Moreover, incorrectly classified samples were mostly assigned to the neighbour classes. Unfortunately, the DenseNet-1024 trained on T-1024-A was unable to recognize samples of the class 0. Finally, the DenseNet-1024 trained on T-1024-B showed similar performance considering the classes 1 to 5 but it also correctly identified samples of the class 0 (Fig 10).

## Discussion

The automatic assessment of cardiomyocyte development stages in confocal microscopy images is a complex task due to the high variability of the cardiomyocytes in the images. We proved the DenseNet topology is suitable for this task. Considering the evaluation results (Table 4), we find the DenseNet-1024 trained on T-1024-B to be ideal for SAACS implementation, as it nearly correctly classified samples of all classes (Fig 10). Although the classes 1 to 4 still contained a number of wrongly classified samples, the errors were acceptable, as a typical error was an assignment of the sample into a neighbouring class. We expect that further improvement of SAACS performance will be possible once SAACS is applied in practice, as SAACS can be retrained on its outputs in semi-supervised manner.

SAACS based on DenseNet-1024, in combination with the automatic high-throughput confocal imaging, opens the possibility to use statistical analysis of large cell populations in the research of the tubular system development or remodelling and loss. This approach has several advantages compared to the conventional methods such as 2D spatial Fourier transform [8–11] and stereological analysis [5]. The 2D spatial Fourier transform is efficient only for late development stages, where discerned patterns of tubules running perpendicular to the long axis of the cell are formed. The stereological analysis can be theoretically applied over a whole range of developmental stages, but it is impractical due to the necessity to analyse hundreds of images by experts in high detail. Our approach allowed studying of the development process in its full range, from the earliest to the latest developmental stages and needed only 90 images assessed by the expert.

The results in Table 4 further show that, compared to the conventional data augmentation techniques (T-512-A and T-1024-A), the augmentation of training sets using manually modified images (T-512-B and T-1024-B) improves performance of both SAACS versions (DenseNet-512 and DenseNet-1024). In our experiments, two new images were created from each source image. As expected, such groups of images emphasize differences between target (Fig 3b)) and non-target objects (Fig 3c)). This approach allowed us to improve correct recognition of class 0 samples which is especially evident in the DenseNet-1024 results (Figs 9 and 10). As this data augmentation technique proved to be highly efficient, we recommend its utilization whenever only a limited set of source images is available.

We observed a marked difference in performance between the DenseNet-512 and the DenseNet-1024 (Table 4), where the DenseNet-1024 showed better performance. The obtained results confirm our hypothesis that the high-resolution images are essential for proper operation of SAACS. We find the explanation for the better performance of SAACS with high-resolution images in the fine pattern of t-tubules, which is one of the most important distinctive characteristics of cardiomyocyte development stages. The need for high-resolution microscopy images was also observed in a study that used a deep ConvNet in digital histopathology images of resolution 224 × 224 px to classify glioblastoma multiform and low-grade glioma [17]. We expect that fine patterns are important in many similar tasks within biology. As the fine patterns disappear while down-sampling the images, high-resolution object images must be used to ensure optimal performance of image categorization systems in cases where the fine pattern is distinctive, even accounting for the higher computational costs in processing large images. We feel the need to highlight this fact since low-resolution images are preferred in practice for their low computational cost. For example, 78 × 78 px object images were used for HEp-2 cell image classification [16], and for classification of red blood cells in sickle cell anemia [23]. The basic prerequisite for the successful use of low-resolution images for object categorization is the existence of discriminatory features in these images. In the cases of the HEp-2 cell [16] and red blood cells images [23], expressive patterns and distorted morphologies, respectively, were the adequate features for the given classification tasks.

## Conclusion

We developed the system for automatic assessment of cardiomyocyte development stages in confocal microscopy images (SAACS). In combination with automatic high-throughput confocal imaging, SAACS will allow the use of statistical analysis for the research on the tubular system development, remodelling and loss. Classification of large cardiomyocyte populations and studying the class distribution in populations under different conditions may become a new approach in the study of structural remodelling associated with physiological or pathological processes, as well as in the development of protocols for the induction of maturation in induced pluripotent cell derived cardiomyocytes.

The development of SAACS was complicated by: a) high variability in the cardiomyocyte shape and structure, and b) lack of source images. To face these issues, we based SAACS on the DenseNet topology and we modified the standard data augmentation technique with manually adjusting source images. As both issues are common in biology, we recommend using the DenseNet and the augmentation technique in biological applications, such as morphology-based cell sorting, identification of regions of interest or projects that today require Citizen science approach. DenseNets should be used instead of the commonly used early deep ConvNets topologies [16, 17, 23] especially if target objects exhibit high variability in their shapes and structures. We also advise to use the manually modified images whenever only a limited collection of source images is available, especially if there is problem with recognition of the object of interest. Considering the fact that the DenseNets can process any type of images (monochromatic, colour, hyperspectral, etc.), DenseNets in combination with the data augmentation technique are ideal means for creation of image categorization systems for various tasks in biology.

## Supporting information

S1 File Implementation of the SAACS in python. Codes that were used for training and evaluation of the DenseNet-512 and DenseNet-1024, including code which delineates class probability graphs.
(RAR)

## References

[1] Bers DM. Calcium fluxes involved in control of cardiac myocyte contraction. Circulation Research. 2000;87(4):275–281. 10.1161/01.RES.87.4.275 10948060

[2] Franzini-Armstrong C. The relationship between form and function throughout the history of excitation–contraction coupling. The Journal of General Physiology. 2018. 10.1085/jgp.201711889 PMC5806676 29317466

[3] Louch WE, Koivumäki JT, P T. Calcium signalling in developing cardiomyocytes: implications for model systems and disease. The Journal of physiology. 2015;593(Pt 5):1047–1063. 10.1113/jphysiol.2014.274712 25641733 PMC4358669

[4] Parikh SS, Blackwell DJ, Gomez-Hurtado N, Frisk M, Wang L, Kim K, et al Thyroid and Glucocorticoid Hormones Promote Functional T-Tubule Development in Human-Induced Pluripotent Stem Cell-Derived Cardiomyocytes. Circulation Research. 2017;121(12):1323–1330. 10.1161/CIRCRESAHA.117.311920 28974554 PMC5722667

[5] Macková K, Zahradníková A, Hot́ka M, Hoffmannová B, Zahradník I, Zahradníková A. Calcium release-dependent inactivation precedes formation of the tubular system in developing rat cardiac myocytes. European Biophysics Journal. 2017;46(8):691–703. 10.1007/s00249-017-1249-z 28913625

[6] High-Throughput Calcium and Contractility System; 2018. Available from: http://www.ionoptix.com/wp-content/uploads/2017/11/multicell_online.pdf.

[7] Klimas A, Ambrosi CM, Yu J, Williams JC, Bien H, Entcheva E. OptoDyCE as an automated system for high-throughput all-optical dynamic cardiac electrophysiology. Nature Communications. 2016;7(11542):1–12. 10.1038/ncomms11542 PMC4866323 27161419

[8] Guo A, Song LS. AutoTT: automated detection and analysis of T-tubule architecture in cardiomyocytes. Biophysical Journal. 2014;106(12):2729–2736. 10.1016/j.bpj.2014.05.013 24940790 PMC4070273

[9] Frisk M, Ruud M, Espe EKS, Aronsen JM, Røe ÅT, Zhang L, et al Elevated ventricular wall stress disrupts cardiomyocyte t-tubule structure and calcium homeostasis. Cardiovascular Research. 2016;112(1):443–451. 10.1093/cvr/cvw111 27226008 PMC5031949

[10] Singh JK, Barsegyan V, Bassi N, Marszalec W, Tai S, Mothkur S, et al T-tubule remodeling and increased heterogeneity of calcium release during the progression to heart failure in intact rat ventricle. Physiological Reports. 2017;5(24):e13540 10.14814/phy2.13540 29279414 PMC5742703

[11] Zhang C, Chen B, Wang Y, Guo A, Tang Y, Khataei T, et al MG53 is dispensable for T-tubule maturation but critical for maintaining T-tubule integrity following cardiac stress. Journal of Molecular and Cellular Cardiology. 2017;112:123–130. 10.1016/j.yjmcc.2017.08.007 28822805 PMC5682927

[12] DiCarlo JJ, Zoccolan D, Rust NC. How does the brain solve visual object recognition? Neuron. 2012;73(3):415–434. 10.1016/j.neuron.2012.01.010 22325196 PMC3306444

[13] LeCun Y, Bengio Y, Hinton G. Deep learning. Nature. 2015;521:436–444. 10.1038/nature14539 26017442

[14] Pinto N, Cox DD, DiCarlo JJ. Why is Real-World Visual Object Recognition Hard? PLOS Computational Biology. 2008;4(1):1–6. 10.1371/journal.pcbi.0040027 PMC2211529 18225950

[15] Lemnaru C, Potolea R. Imbalanced Classification Problems: Systematic Study, Issues and Best Practices In: Enterprise Information Systems. Berlin, Heidelberg: Springer Berlin Heidelberg; 2012 p. 35–50.

[16] Gao Z, Wang L, Zhou L, Zhang J. HEp-2 Cell Image Classification With Deep Convolutional Neural Networks. IEEE Journal of Biomedical and Health Informatics. 2017;21(2):416–428. 10.1109/JBHI.2016.2526603 26887016

[17] Xu Y, Jia Z, Ai Y, Zhang F, Lai M, Chang EI. Deep convolutional activation features for large scale Brain Tumor histopathology image classification and segmentation. In: 2015 IEEE International Conference on Acoustics, Speech and Signal Processing (ICASSP); 2015. p. 947–951.

[18] Tajbakhsh N, Shin JY, Gurudu SR, Hurst RT, Kendall CB, Gotway MB, et al Convolutional Neural Networks for Medical Image Analysis: Full Training or Fine Tuning? IEEE Transactions on Medical Imaging. 2016;35(5):1299–1312. 10.1109/TMI.2016.2535302 26978662

[19] Zeiler MD, Fergus R. Visualizing and Understanding Convolutional Networks In: Computer Vision—ECCV 2014. Cham: Springer International Publishing; 2014 p. 818–833.

[20] Simonyan K, Zisserman A. Very Deep Convolutional Networks for Large-Scale Image Recognition. CoRR. 2014;abs/1409.1556.

[21] Krizhevsky A, Sutskever I, Hinton GE. ImageNet Classification with Deep Convolutional Neural Networks. Communications of the ACM. 2017;60(6):84–90. 10.1145/3065386

[22] Bengio Y, Simard P, Frasconi P. Learning long-term dependencies with gradient descent is difficult. IEEE Transactions on Neural Networks. 1994;5(2):157–166. 10.1109/72.279181 18267787

[23] Xu M, Papageorgiou DP, Abidi SZ, Dao M, Zhao H, Karniadakis GE. A deep convolutional neural network for classification of red blood cells in sickle cell anemia. PLOS Computational Biology. 2017;13(10):1–27. 10.1371/journal.pcbi.1005746 PMC5654260 29049291

[24] Szegedy C, Liu W, Jia Y, Sermanet P, Reed S, Anguelov D, et al. Going deeper with convolutions. In: 2015 IEEE Conference on Computer Vision and Pattern Recognition (CVPR); 2015. p. 1–9.

[25] Szegedy C, Vanhoucke V, Ioffe S, Shlens J, Wojna Z. Rethinking the Inception Architecture for Computer Vision. In: 2016 IEEE Conference on Computer Vision and Pattern Recognition (CVPR); 2016. p. 2818–2826.

[26] Srivastava RK, Greff K, Schmidhuber J. Training Very Deep Networks. In: Proceedings of the 28th International Conference on Neural Information Processing Systems—Volume 2. NIPS’15. Cambridge, MA, USA: MIT Press; 2015. p. 2377–2385.

[27] He K, Zhang X, Ren S, Sun J. Deep Residual Learning for Image Recognition. In: 2016 IEEE Conference on Computer Vision and Pattern Recognition (CVPR); 2016. p. 770–778.

[28] He K, Zhang X, Ren S, Sun J. Identity Mappings in Deep Residual Networks In: Computer Vision—ECCV 2016. Cham: Springer International Publishing; 2016 p. 630–645.

[29] Huang G, Sun Y, Liu Z, Sedra D, Weinberger KQ. Deep Networks with Stochastic Depth In: Leibe B, Matas J, Sebe N, Welling M, editors. Computer Vision—ECCV 2016. Cham: Springer International Publishing; 2016 p. 646–661.

[30] Han D, Kim J, Kim J. Deep Pyramidal Residual Networks. In: 2017 IEEE Conference on Computer Vision and Pattern Recognition (CVPR); 2017. p. 6307–6315.

[31] Huang G, Liu Z, v d Maaten L, Weinberger KQ. Densely Connected Convolutional Networks. In: 2017 IEEE Conference on Computer Vision and Pattern Recognition (CVPR); 2017. p. 2261–2269.

[32] Yu Z, Li T, Luo G, Fujita H, Yu N, Pan Y. Convolutional networks with cross-layer neurons for image recognition. Information Sciences. 2018;433-434:241–254. 10.1016/j.ins.2017.12.045

[33] Wang S, Liu W, Wu J, Cao L, Meng Q, Kennedy PJ. Training deep neural networks on imbalanced data sets. In: 2016 International Joint Conference on Neural Networks (IJCNN); 2016. p. 4368–4374.

[34] Škrabánek P, Doležel P. On reporting performance of binary classifiers. Scientific Papers of the University of Pardubice, Series D: Faculty of Economics and Administration. 2017;24(41):181–192.

[35] Zahradníková jr A, Škrabánek P. Confocal Microscopy Images of Cardiomyocyte Development Stages; 2019. Available from: 10.6084/m9.figshare.8063369. PMC6542571 31145728

[36] Ioffe S, Szegedy C. Batch normalization: Accelerating deep network training by reducing internal covariate shift. In: Proceedings of the 32nd International Conference on Machine Learning, ICML 2015. vol. 37 of Proceedings of Machine Learning Research. PMLR; 2015. p. 448–456.

[37] Sokolova M, Lapalme G. A systematic analysis of performance measures for classification tasks. Information Processing & Management. 2009;45(4):427–437. 10.1016/j.ipm.2009.03.002

[38] Ruder S. An overview of gradient descent optimization algorithms. CoRR. 2016;abs/1609.04747.

[39] Abadi M, Agarwal A, Barham P, Brevdo E, Chen Z, Citro C, et al. TensorFlow: Large-Scale Machine Learning on Heterogeneous Systems; 2015. Available from: https://www.tensorflow.org/.

